# Supplementary material for: Mixed-methods evaluation of a group psychosocial intervention for refugee, migrant and host community women in Ecuador and Panamá: Results from the Entre Nosotras cluster randomized feasibility trial
Source: Glob Ment Health (Camb). 2023 Jul 19;10:e42. doi: 10.1017/gmh.2023.37 (PMC10579653; doi:10.1017/gmh.2023.37)
Supplement: Supplementary file 1 [file S2054425123000377sup001.docx]

**Supplemental Files**

**Supplemental Table. 1. Overview of the Entre Nosotras intervention**

| **Session** | **Objectives** | **Main Components** |
| --- | --- | --- |
| 1. Building trust, security & connection | - Build trust and group identity - Collectively set group expectations, rules, and goals - Introduce concepts of coping and grounding (in enhanced condition only) | - Icebreaker activity (locally developed) - Activities to build group identity and trust (locally developed) - Community mapping to strengthen connectedness and solidarity (Metodologías comunitarias participativas) - Coping and grounding (Self Help Plus) |
| 2. Psychosocial wellbeing | - Define the concept of psychosocial wellbeing and its dimensions - Describe the factors that contribute to psychosocial wellbeing, including the impacts of adversity - Practice grounding as a coping skill (in enhanced condition only) | - Group check-in and icebreaker activity (locally developed) - Psychoeducation (HIAS Community-Based MHPSS curriculum; Psychological First Aid) - Understanding adversity (Problem Management Plus) - Defining and conceptualizing wellbeing (locally developed) - Grounding (Self Help Plus) |
| 3. Gender, gender-based violence, and safety | - Reflect on concepts related to gender, wellbeing, and protection - Identify resources to promote the safety and wellbeing of women - Introduce unhooking from difficult thoughts and feelings as well as acting according to your values as coping skills (in enhanced condition only) | - Group check-in and icebreaker activity (locally developed) - Understanding the relationship between gender, sense of security, and psychosocial wellbeing (locally developed) - Designing a protection plan (Metodologías comunitarias participativas; Community Action Cycle) - Unhooking from difficult thoughts and feelings, acting according to your values (Self Help Plus) |
| 4. Strengths and psychosocial resources | - Identify social and psychological strategies and resources to support safety and psychosocial wellbeing - Mobilize peer support - Revisit acting on your values and introduce being kind as coping skills (in enhanced condition only) | - Group check-in and icebreaker activity (locally developed) - Strengthening social support (HIAS Community-Based MHPSS curriculum) - Community mapping and protection plan (Metodologías comunitarias participativas; Community Action Cycle) - Acting according to your values and being kind (Self Help Plus) |
| 5. Community resources | - Identify community resources that promote protection, safety, and wellbeing - Strengthen community support networks - Introduce making room as a coping skill (in enhanced condition only) - Generate a group support and action plan for the future | - Group check-in and icebreaker activity (locally developed) - Making space (Self Help Plus) - Thinking about the future and planning new actions (Metodologías comunitarias participativas; Community Action Cycle) - Closing activities (locally developed) |

**Supplemental Table 2. Baseline correlates of attrition (complete both follow-up assessments; n=64, 28.4% missed at least 1 follow-up) and intervention completion (4+ sessions; n=112, 49.8% completed 4+ sessions)**

|  | **Assessment completion**  **OR (95% CI)** | **Intervention completion**  **OR (95% CI)** |
| --- | --- | --- |
| ***Demographic Characteristics*** |  |  |
| Age (in years) | 0.99 (0.96, 1.01) | 1.05 (1.02, 1.07) |
| Site |  |  |
| Guayaquil | REF | REF |
| Panama | 0.54 (0.27, 1.08) | 2.33 (1.22, 4.46) |
| Tulcan | 0.52 (0.25, 1.08) | 1.53 (0.79, 2.97) |
| Education |  |  |
| *Less than primary* | REF | REF |
| *Completed primary school* | 0.30 (0.06, 1.43) | 1.08 (0.28, 4.13) |
| *Completed secondary school* | 0.53 (0.16, 1.76) | 1.17 (0.37, 3.69) |
| *College degree* | 0.99 (0.29, 3.42) | 0.97 (0.29, 3.27) |
| *Other* | 0.64 (0.13, 3.20) | 2.92 (0.59, 14.33) |
| Employment |  |  |
| *Unemployed or Housewife* | REF | REF |
| *Informal work* | 1.88 (0.97, 3.62) | 0.55 (0.30, 1.01) |
| *Part-time formal work* | 1.72 (0.54, 5.47) | 0.53 (0.18, 1.57) |
| *Full-time formal work* | 0.85 (0.67, 5.11) | 0.79 (0.31, 2.04) |
| Any employment |  |  |
| *Unemployed or housewife* | REF | REF |
| *Employed* | 1.87 (1.04, 3.37) | 0.59 (0.35, 1.01) |
| Nationality |  |  |
| *Colombian* | REF | REF |
| *Ecuadorian* | 0.38 (0.11, 1.40) | 2.35 (0.81, 6.84) |
| *Venezuelan* | 0.98 (0.43, 2.24) | 0.79 (0.37, 1.68) |
| *Other (Cuban, Dominican, Honduran, Nicaraguan, Salvadoran, Mixed)* | 1.15 (0.31, 4.24) | 0.82 (0.24, 2.80) |
| Community |  |  |
| *Migrant* | REF | REF |
| *Host* | 0.38 (0.13, 1.14) | 2.85 (1.20 6.79) |
| How long have you lived in your current community |  |  |
| *Less than one year* | REF | REF |
| *1-3 years* | 0.71 (0.35, 1.45) | 1.08 (0.54, 2.14) |
| *More than 3 years* | 0.43 (0.20, 0.93) | 2.32 (1.14, 4.69) |
| Primary reason for moving to study community |  |  |
| *Born in the community* | No LTF | All completed |
| *Migrated for work and/or more opportunities* | REF | REF |
| *Migrated for family reasons* | 0.35 (0.10, 1.19) | 5.50 (1.36, 22.22) |
| *Migrated due to political violence or armed conflict* | 0.20 (0.05, 0.82) | 2.75 (0.63, 12.08) |
| *Migrated due to economic problems* | 0.26 (0.08, 0.84) | 2.83 (0.74, 10.87) |
| *Other reasons* | 0.41 (0.10, 1.75) | 4.13 (0.84, 20.28) |
| Enhanced study condition (REF = standard) | 1.65 (0.91, 2.98) | 0.38 (0.22, 0.65) |
| ***Mental health and psychosocial outcomes at baseline*** |  |  |
| Psychological distress (Kessler 6) | 1.04 (0.95, 1.14) | 0.91 (0.84, 0.99) |
| Life Satisfaction (PWI Single Item) | 1.05 (0.91, 1.20) | 0.96 (0.85, 1.09) |
| Psychosocial Wellbeing (PWI Total) | 1.03 (1.003, 1.06) | 0.98 (0.97, 1.01) |
| Community Connectedness (PWI Subscale) | 1.06 (0.93, 1.20) | 0.99 (0.88, 1.11) |
| Sense of Safety (PWI Subscale) | 1.04 (0.92, 1.17) | 0.97 (0.87, 1.08) |
| Coping (Brief Cope) | 0.98 (0.95, 1.02) | 1.01 (0.98, 1.04) |
| Problem-focused coping (Brief Cope) | 0.98 (0.92, 1.05) | 1.02 (0.96, 1.09) |
| Emotion-focused coping (Brief Cope) | 0.96 (0.92, 1.02) | 1.02 (0.97, 1.07) |
| Avoidant coping (Brief Cope) | 0.99 (0.92, 1.07) | 1.02 (0.95, 1.09) |
| Social Support (Oslo-3) | 0.97 (0.86, 1.09) | 1.09 (0.98, 1.21) |
| Functional Impairment (WHODAS) | 1.00 (0.96, 1.05) | 0.98 (0.94, 1.02) |

**Supplemental Figure 1. Attendance in overall sample by condition**

**Supplemental Figure 2. Attendance by study communit**

**Supplemental Table 3. Psychometric performance of outcome variables at baseline**

|  | **Kessler-6** | | **Personal Wellbeing Index** | | **Brief Cope** | | | **Oslo Social Support Scale** | **WHODAS** | |
| --- | --- | --- | --- | --- | --- | --- | --- | --- | --- | --- |
| **Internal Consistency** | | | | | | | | | | |
| *Cronbach’s Alpha* | 0.483 | | 0.781 | | 0.784 | | | 0.539 | 0.842 | |
| *Any items that should be deleted* | #5/E: ‘feeling that everything was an effort’ (alpha if item deleted=0.594) | | None | | None | | | None | None | |
| **Confirmatory Factor Analysis** | | | | | | | | | | |
|  | 6-item | 5-item | 8-item | 8-item | 28-item | 28-item | 28-item | 3-item | 12-item | 12-item |
| *Number of factors* | 1 | 1 | 1 | 1 | 1 | 3 | 3 | 1 | 1 | 1 |
| *Chi-squared*  *(model vs. saturated)* | 11.3 (p=0.258) | 6.1 (p=0.297) | 41.8 (p=0.003) | 29.21 (p=0.063) | 1157.04 (p<.001) | 1061.50 (p<.001) | 487.2 (p<.001) | -- | 311.1 (p<.001) | 95.0 (p<.001) |
| *CFI* | 0.977 | 0.989 | 0.941 | 0.972 | 0.370 | 0.442 | 0.875 | 1.000 | 0.699 | 0.946 |
| *TLI* | 0.961 | 0.977 | 0.917 | 0.959 | 0.319 | 0.392 | 0.855 | 1.000 | 0.632 | 0.927 |
| *RMSEA* | 0.034 | 0.031 | 0.070 | 0.049 | 0.104 | 0.098 | 0.048 | 0.000 | 0.147 | 0.065 |
| *SRMR* | 0.040 | 0.032 | 0.050 | 0.042 | 0.108 | 0.105 | 0.075 | 0.000 | 0.096 | 0.057 |
| *Modifications to factor structure* | None | Removed item ‘e’: ‘feeling that everything was an effort’ | None | Added covariance between ‘standard of living’ and ‘health’ satisfaction | None | Standard 3-factor solution | Covariances and cross-loadings (see notes) | None | None | Covariances (see notes) |

Brief Cope Revised Model:

- Covariances between items 18/28, 4/11, 13/26, 5/15, 22/27, 12/17, 10/23, 3/8, 7/11, 10/5, 2/1, 9/21, 20/24, 1/19, 10/15, 19/20
- Cross loadings: items 1 (‘I’ve been looking for something good in what is happening’) and 26 (‘I’ve been blaming myself for things that happened’) on avoidant, problem, and emotion-focused coping

WHODAS Revised Model:

- Covariances between items 8/9, 1/7, 1/10, 10/11, 6/11

**Supplemental Table 4. Percent of intervention components delivered with full fidelity by session and site**

| **Session Fidelity** |  | **Overall** |  | **Site**  (% of components delivered with full fidelity) | | |
| --- | --- | --- | --- | --- | --- | --- |
|  |  |  |  | **Guayaquil** | **Panama** | **Tulcan** |
| *External Raters* | | | | | | |
| 1 |  | 85.7% |  | 76.2% | 91.3% | 88.8% |
| 2 |  | 96.4% |  | 98.0% | 96.1% | 95.2% |
| 3 |  | 96.8% |  | 97.6% | 97.5% | 95.2% |
| 4 |  | 91.5% |  | 97.6% | 89.2% | 88.1% |
| 5 |  | 91.4% |  | 83.8% | 97.6% | 92.9% |
| All |  | 92.3% |  | 91.3% | 94.4% | 92.0% |
| *Self-Rated* | | | | | | |
| 1 |  | 87.3% |  | 93.5% | 89.6% | 66.7% |
| 2 |  | 75.1% |  | 50.0% | 92.9% | 44.4% |
| 3 |  | 80.6% |  | 83.3% | 91.3% | 35.0% |
| 4 |  | 79.8% |  | 79.2% | 85.0% | 70.0% |
| 5 |  | 87.5% |  | 91.7% | 100.0% | 58.3% |
| All |  | 72.2% |  | 80.7% | 91.2% | 57.1% |
